# Indicadores do uso de medicamentos na atenção primária de saúde: uma revisão sistemática

**DOI:** 10.26633/RPSP.2017.132

**Published:** 2017-11-11

**Authors:** André Santos da Silva, Gabriella de Alcantara Maciel, Luciane Soares de Lima Wanderley, Almir Gonçalves Wanderley

**Affiliations:** 1 Universidade Federal do Vale do São Francisco Colegiado de Farmácia Petrolina (PE) Brasil Universidade Federal do Vale do São Francisco, Colegiado de Farmácia, Petrolina (PE), Brasil.; 2 Centro Universitário Tabosa de Almeida Curso de Farmácia Caruaru (PE) Brasil Centro Universitário Tabosa de Almeida, Curso de Farmácia. Caruaru (PE), Brasil.; 3 Universidade Federal de Pernambuco Departamento de Enfermagem Recife (PE) Brasil Universidade Federal de Pernambuco, Departamento de Enfermagem, Recife (PE), Brasil.; 4 Universidade Federal de Pernambuco Departamento de Fisiologia e Farmacologia Recife (PE) Brasil Universidade Federal de Pernambuco, Departamento de Fisiologia e Farmacologia, Recife (PE), Brasil.

**Keywords:** Uso de medicamentos, atenção primária à saúde, indicadores, revisão sistemática, Drug utilization, primary health care, indicators, review, systematic, Utilización de medicamentos, atención primaria de salud, indicadores, revisión sistemática

## Abstract

**Objective.:**

To analyze the rational use of medicines in the context of primary health care (PHC) according to the indicators recommended by the World Health Organization (WHO).

**Method.:**

A systematic review of the literature was performed following PRISMA guidelines to synthesize the evidence produced by the application of drug use indicators in PHC in the period from 2011 to 2016. The following databases were searched: PudMed, SciELO and Google Scholar, Virtual Health Library/BIREME, and *Portal de Periódicos CAPES*, using the keywords “World Health Organization,” “indicators,” “drug utilization,” and “rational use of drugs” in Portuguese and English. Original articles describing studies performed at the PHC level, using at least one of the three sets of indicators (prescription, service-related, or health care) were included.

**Results.:**

Of the 16 studies included, 56.2% were prospective, 37.5% were developed at a local level, 62.5% employed convenience sampling, 56.2% lasted up to 6 months, and 43.8% were performed in Brazil. Prescription indicators were used most (87.5%), followed by service-related indicators (37.5%) and health care indicators (31.3%). None of the scenarios described in the articles fully met the WHO recommendations. The most frequent interventions suggested to resolve the problems related to the rational use of medications included continuing education for rational prescription (56.3%), use of updated lists of essential medicines, including generic drug names and reflecting the needs of the population (31.3%), and implementation of clinical protocols to standardize therapeutic management (31.3%).

**Conclusions.:**

Application of the WHO indicators revealed irrational practices of drug use in PHC in several countries.

## RESUMEN

**Objetivo.:**

Analizar el uso racional de medicamentos en la atención primaria de salud teniendo en cuenta los indicadores recomendados por la Organización Mundial de la Salud.

**Métodos.:**

Se llevó a cabo una revisión sistemática de la bibliografía con base en la recomendación PRISMA para recopilar evidencia relativa a la utilización de indicadores de uso de medicamentos en la atención primaria entre el 2011 y el 2016. Se hicieron búsquedas en las bases PudMed, SciELO, Google Académico, la Biblioteca Virtual en Salud (BVS) y el Portal de Periódicos CAPES con los términos clave “Organización Mundial de la Salud”, “indicadores”, “uso de medicamentos” y “uso racional de medicamentos”, tanto en portugués como en inglés. Se incluyeron artículos originales sobre estudios en la atención primaria que usaban al menos uno de los tres conjuntos de indicadores (prescripción, servicio o asistencia).

**Resultados.:**

Se incluyeron 16 publicaciones: 56,2 % eran estudios prospectivos, 37,5 % eran del ámbito local, 62,5 % usaron muestreo por conveniencia, 56,2 % tuvieron una duración máxima de seis meses y 43,8 % procedían de Brasil. Los indicadores más utilizados fueron los de la prescripción (87,5 %), seguidos de los de servicio (37,5 %) y asistencia (31,3 %). Ninguno de los escenarios descriptos en los estudios cumplía plenamente las recomendaciones de la OMS. Para resolver los problemas de uso racional de medicamentos se plantearon sobre todo intervenciones sobre educación continua en materia de prescripción racional (56,3 %), uso de la lista actualizada de medicamentos esenciales (31,3 %) que incluyera el nombre genérico y atendiera a las necesidades de la población y aplicación de protocolos clínicos para estandarizar las conductas terapéuticas (31,3 %).

**Conclusiones.:**

Los indicadores de la OMS revelaron prácticas irracionales de consumo de medicamentos en atención primaria de salud en varios países.

## RESUMO

**Objetivo.:**

Analisar o uso racional de medicamentos na atenção primária em saúde (APS) a partir dos indicadores preconizados pela Organização Mundial da Saúde (OMS).

**Métodos.:**

Foi feita uma revisão sistemática da literatura com base na recomendação PRISMA para sintetizar evidências relacionadas à utilização dos indicadores de uso de medicamentos na APS no período de 2011 a 2016. As seguintes bases foram consultadas: PudMed, SciELO e Google Acadêmico, Biblioteca Virtual em Saúde (BVS) e Portal de Periódicos CAPES, utilizando as palavras-chave “Organização Mundial da Saúde”, “indicadores”, “uso de medicamentos” e “uso racional de medicamentos” em português e inglês. Foram incluídos artigos originais sobre estudos na APS que utilizassem pelo menos um dos três conjuntos de indicadores (prescrição, serviço ou assistência).

**Result ados.:**

Foram incluídas 16 publicações, sendo 56,2% estudos prospectivos, 37,5% de nível local, 62,5% com amostragem por conveniência, 56,2% com duração de até 6 meses e 43,8% provenientes do Brasil. Os indicadores mais utilizados foram os da prescrição (87,5%), seguidos dos indicadores sobre o serviço (37,5%) e assistência (31,3%). Nenhum dos cenários relatados nos estudos atendeu plenamente as recomendações da OMS. As intervenções mais sugeridas para resolver os problemas de uso racional de medicamentos incluíram educação continuada para prescrição racional (56,3%), uso da lista de medicamentos essenciais atualizada, incluindo o nome genérico dos medicamentos e atendendo as necessidades da população (31,3%), e implementação de protocolos clínicos para padronizar condutas terapêuticas (31,3%).

**Conclusões.:**

Os indicadores da OMS revelaram práticas irracionais de uso de medicamentos na APS em diversos países.

O medicamento, quando adequadamente utilizado, é um recurso terapêutico essencial para as políticas de saúde. Por outro lado, o seu uso inadequado é um grave problema de saúde pública. Em todo o mundo, as estimativas mostram que mais da metade de todos os medicamentos são prescritos, dispensados ou vendidos de forma inadequada, e que aproximadamente 50% dos usuários não utilizam os medicamentos corretamente (1). O estudo de Kripalani et al. (2) mostrou que, dentre os indivíduos que utilizam os medicamentos de forma inadequada, 22,9% podem sofrer implicações de saúde consideradas graves; no caso dos cardiopatas, o risco de morte na presença de uso inadequado de medicamentos é de 1,8% (2).

Cada vez mais, os medicamentos vêm sendo tratados como bens de consumo, e não como bens de serviço básicos para auxiliar na promoção da saúde. O estímulo ao consumo de medicamentos vem aumentando e induzindo o seu uso irracional; são exemplos a polimedicação (3), a falta de prescrição de acordo com as diretrizes clínicas (4) e o uso inadequado de antimicrobianos (5). Os motivos que levam ao uso irracional de medicamentos são inúmeros, destacando-se o número excessivo de produtos farmacêuticos no mercado, a facilidade no acesso aos medicamentos, a prática da automedicação, a falta de informações aos usuários, as prescrições ilegíveis ou incompletas, a disponibilidade ainda insuficiente de diretrizes clínicas, a divulgação de informações inapropriadas sobre os medicamentos e a propaganda de medicamentos (6).

O uso racional de medicamentos foi definido pela Organização Mundial da Saúde (OMS) como o recebimento e a utilização de medicamentos apropriados para a situação clínica, em doses que satisfaçam as necessidades do indivíduo, por um período adequado e ao menor custo possível para o próprio paciente e sua comunidade (7). Conforme a OMS, a promoção do uso racional de medicamentos é um componente importante nas políticas nacionais de medicamentos (8). Além disso, são pré-requisitos para o uso racional de medicamentos a implementação da Lista Local de Medicamentos Essenciais (LLME), o registro e o uso de medicamentos genéricos, a elaboração de um formulário terapêutico nacional, a formação de recursos humanos voltados para o gerenciamento do uso de medicamentos e a realização de campanhas educativas e de ações de farmacovigilância, entre outros (9).

Em 1993, a OMS, em colaboração com a Rede Internacional para o Uso Racional de Drogas (INRUD), apresentou um conjunto de indicadores básicos, denominados “indicadores do uso de medicamentos”, com o objetivo de delinear métodos para a coleta de dados e quantificar o desempenho dos serviços de saúde em três grandes áreas relacionadas ao uso racional de medicamentos na atenção primária à saúde (APS): área da prescrição, área da assistência ao paciente e área do serviço de saúde (10). São exemplos dos indicadores da prescrição: número médio de medicamentos prescritos por consulta; porcentagem de medicamentos prescritos por nome genérico; porcentagem de consultas nas quais se prescreve antibiótico; porcentagem de consultas nas quais se prescreve medicamento injetável; e porcentagem de medicamentos prescritos que estão presentes na lista local de medicamentos essenciais. Para os indicadores da assistência ao paciente, são exemplos o tempo médio de consulta; tempo médio de dispensação; porcentagem de medicamentos corretamente etiquetados; porcentagem de medicamentos prescritos que realmente são dispensados; e porcentagem de pacientes que conhecem a dose correta. Por fim, são exemplos dos indicadores sobre o serviço de saúde a disponibilidade de cópias da lista ou formulário de medicamentos essenciais; e a disponibilidade dos medicamentos essenciais ou medicamentos-chave no próprio serviço onde são prescritos (10).

Em termos gerais, esses indicadores são medidas objetivas de síntese ou estatísticas. Tomadas em conjunto, essas medidas permitem descrever a racionalidade do uso de medicamentos em grandes e pequenos centros de saúde, regiões ou países. Tais indicadores podem ainda ser aplicados de forma padronizada sem a necessidade de treinamento especial da equipe ou grandes recursos (10).

O termo APS, internacionalmente adotado, representa uma estratégia de organização da atenção à saúde de forma regionalizada, contínua e sistematizada voltada para o atendimento das necessidades básicas de saúde de uma população, integrando ações preventivas e curativas, bem como a atenção a indivíduos e comunidades (11). Considerando a importância dos medicamentos para a saúde das populações, estudos com foco nos indicadores de uso de medicamentos podem auxiliar nas tomadas de decisão, sobretudo quando fundamentados em informações de produto, serviço ou processo. Assim, o presente artigo apresenta uma revisão sistemática acerca das evidências disponíveis na literatura sobre a utilização dos indicadores do uso de medicamentos na APS no período de 2011 a 2016.

## MATERIAIS E MÉTODOS

Inicialmente, realizou-se uma busca na literatura para verificar a existência de revisões sistemáticas anteriores tratando do mesmo tema e com o mesmo objetivo. Como nenhum estudo nessas perspectivas foi identificado, prosseguiu-se com a presente revisão sistemática.

A revisão sistemática da literatura baseou-se nas recomendações propostas pelo guia *Preferred Reporting Items for Systematic Reviews and Meta-Analyses* (PRISMA) (12). Entretanto, optamos por limitar a busca às bases eletrônicas, sem busca manual em outras fontes. Foram consultadas as bases PubMed, SciELO, Google Acadêmico, Biblioteca Virtual em Saúde - BVS/BIREME e o Portal de Periódicos CAPES/MEC durante o período de 19 a 24 de novembro de 2016, usando as seguintes palavras-chave em inglês e em português, combinadas com operadores booleanos: (“Organização Mundial da Saúde” OR “OMS” OR “World Health Organization” OR “WHO”) AND (“Indicadores” OR “Indicators”) AND (“Drug Utilization” OR “Rational Use of Drugs” OR “Uso de Medicamentos” OR “Uso Racional de Medicamentos”). Esses termos foram selecionados no vocabulário estruturado dos Descritores em Ciências da Saúde (DeCS) (http://decs.bvs.br/ ), criado pela BIREME e desenvolvido a partir dos *Medical Subject Headings* (MeSH), da *U.S. National Library of Medicine.*

Os títulos e resumos dos artigos identificados pela estratégia de busca inicial foram avaliados de forma independente por dois autores (A.S.S. e G.A.M.) seguindo critérios de inclusão e exclusão. Estudos duplicados foram removidos e as diferenças foram resolvidas por consenso. Quando o título ou o resumo não indicavam claramente se um artigo deveria ser selecionado, o texto completo foi obtido e lido para determinar se satisfazia todos os critérios. Os artigos considerados relevantes na primeira triagem foram recuperados e selecionados para elegibilidade. Não houve busca manual nas referências dos artigos selecionados.

### Critérios de inclusão e exclusão

Os critérios de inclusão e exclusão foram definidos com base na pergunta norteadora deste estudo: verificar, a partir dos indicadores do uso de medicamentos preconizados pela OMS, se os serviços de APS vêm alcançando desfechos positivos para o uso racional de medicamentos.

Desse modo, os critérios de inclusão foram: ser artigo original completo (com disponibilidade de texto integral), escrito em inglês, espanhol ou português sobre estudo desenvolvido em serviço de APS; ter população-alvo sem particularidade de faixa etária, gênero ou condição de saúde; utilizar pelo menos um dos três conjuntos de indicadores do uso de medicamentos desenvolvidos pela OMS; e ter sido publicado de 2011 a 2016. Foram excluídos da revisão sistemática: artigos de revisão, resumos, teses e outras monografias; estudos desenvolvidos exclusivamente em serviço de atenção terciária à saúde (incluindo hospitais); estudos com população específica, pediátrica, geriátrica, ou gestantes, ou relacionados com doenças particulares, bem como validações de instrumento; estudos que utilizaram outros métodos para descrever ou mensurar o uso racional de medicamentos ao invés dos indicadores da OMS/INRUD; estudos que não forneceram detalhes suficientes em suas metodologias e resultados para responder a pergunta deste estudo.

No caso de estudos que obtiveram resultados em diferentes níveis de atenção saúde, apenas os resultados relativos à APS foram considerados. Para artigos que apresentaram resultados de indicadores em diferentes localidades ou regiões, foram verificadas e utilizadas as médias desses valores.

### Extração de dados

Os dados dos artigos incluídos foram extraídos e resumidos em uma tabela padronizada que incorporou as seguintes informações: nome do primeiro autor e ano de publicação, país, nível de estudo (local, distrital, provincial ou nacional), localização, duração, objetivo, desenho, tipo de amostragem, número amostral, indicadores utilizados, resultados e conclusões. Essas extrações foram realizadas de forma independente por dois autores (A.S.S., G.A.M.) e as diferenças foram resolvidas por consenso.

### Análise de dados

Foi realizada uma revisão sistemática descritiva, por isso sem metanálise. O estudo tratou os resultados dos indicadores obtidos nos artigos com a mesma importância, sem levar em consideração o tamanho amostral e a variância. Os indicadores de uso de medicamentos foram identificados e seus resultados tabulados no Microsoft Office Excel, versão 2010. Os resultados foram comparados com os valores de referência recomendados pela OMS e verificados quanto à adequação ao uso racional de medicamentos.

## RESULTADOS

**FIGURA 1:**
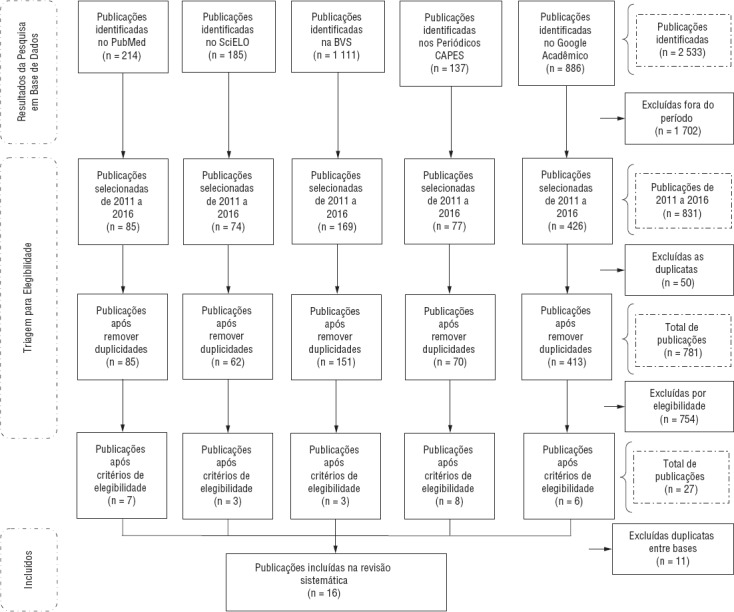
Identificação e seleção dos artigos para revisão sistemática sobre uso racional de medicamentos na atenção primária à saúde, 2011 a 2016

Foram identificadas 2 533 publicações considerando as cinco bases de dados utilizadas. Dessas publicações, 1 752 foram excluídas por estarem fora do período de 2011 a 2016, bem como repetidas dentro da mesma base de dados. Das 781 publicações restantes identificadas, 754 foram eliminadas após o rastreio dos critérios de elegibilidade. Vinte e sete publicações foram eleitas, porém 11 delas foram excluídas por terem duplicatas entre as bases de dados. Assim, foram selecionadas 16 publicações (13 28) que atendiam os critérios de inclusão (figura 1).

Em relação à distribuição geográfica das publicações incluídas na pesquisa, oito (50,0%) foram provenientes de países da América do Sul, cinco (31,2%) da Ásia e três (18,8%) da África. O desenho foi prospectivo em nove (56,2%) publicações e retrospectivo em sete (43,8%). Os indicadores mais utilizados foram os de prescrição, em 14 (87,5%) publicações, seguidos dos indicadores sobre o serviço, com seis estudos (37,5%), e dos indicadores de assistência, com cinco estudos (31,3%). O detalhamento das publicações analisadas está apresentado na tabela 1.

Os resultados relativos aos indicadores do uso de medicamentos da OMS empregados nas publicações estão resumidos na tabela 2. A tabela 3 apresenta intervenções sugeridas pelos autores das publicações com o intuito de resolver problemas que levam ao uso inadequado dos medicamentos. Das recomendações mais citadas, a promoção da prescrição racional por meio da educação continuada dos profissionais de saúde e prescritores obteve a maior frequência (56,3%). Em segundo lugar, com a mesma frequência (31,3%), estiveram a recomendação de uma LLME atualizada, com divulgação pelo nome genérico dos medicamentos e revisão para atender as necessidades da população, e a implementação de protocolos clínicos para padronizar condutas terapêuticas (31,3%). Além disso, foi sugerida a necessidade de mais estudos que investiguem as razões do uso inadequado (18,7%). Outras sugestões incluíram a necessidade de fortalecer as autoridades reguladoras, inserir o farmacêutico na equipe multiprofissional, melhorar a gestão da assistência farmacêutica e promover mais estudos com indicadores (12,5%).

## DISCUSSÃO

Os 16 estudos analisados nesta revisão mostraram uma tendência de maior utilização dos indicadores da OMS/INRUD por parte de países em desenvolvimento ou emergentes. Este resultado era esperado, uma vez que, dos mais de 30 países que aceitam esses indicadores como método padrão, a maioria é de países em desenvolvimento (29). Por outro lado, estudos voltados para a farmacoepidemiologia são ainda escassos em países emergentes. Dessa forma, a falta de informações sobre o consumo e a prescrição de medicamentos no país pode levar à inadequação de sua utilização.

Neste estudo, o grupo mais utilizado de indicadores da OMS/INRUD foi o da prescrição, possivelmente pela facilidade de aplicação que pode ser feita de forma retrospectiva, utilizando arquivos da própria unidade de saúde, o que reduz o tempo e o custo de pesquisa. Outra hipótese está ligada ao próprio fato da prescrição irracional. Embora ocorra em todo o mundo (30), a prescrição irracional se agrava em países de baixa renda e, portanto, precisa ser avaliada, uma vez que vem sendo considerada a principal causa do uso irracional de medicamentos.

Observa-se que a causa do uso não racional de medicamentos é multifatorial. Desse modo, torna-se necessária a obtenção de informações relativas a produto, serviço e processo. A utilização dos indicadores básicos da OMS possibilita, de forma conjunta, descrever com qualidade a racionalidade do uso de medicamentos (10).

### Indicadores da prescrição

O número médio de medicamentos prescritos por consulta observado nas publicações avaliadas esteve acima do preconizado pela OMS em 13 dos 14 estudos que utilizaram indicadores de prescrição (13 22, 24 28). Embora esse resultado não satisfaça a prática do uso racional de medicamentos, vale ressaltar que o indicador tem como objetivo detectar o grau de polimedicação ou polifarmácia; portanto, a definição utilizada é de ≥ 2 medicamentos. Entretanto, a meta de < 2 medicamentos por consulta pode estar subestimada em algumas situações (31, 32). Um estudo anterior (3) utilizou como definição de polimedicação o uso concomitante de cinco ou mais medicamentos. Assim, caso a OMS elegesse o valor de < 5 medicamentos por consulta, apenas um estudo estaria fora do recomendado para atender a prática do uso racional de medicamentos. Portanto, é necessário revisitar o parâmetro adotado pela OMS ou o próprio indicador, o qual poderia ter como proposta avaliar o número total de medicamentos prescritos ao paciente, e não tão somente por consulta, buscando assim adequá-lo à realidade atual.

A porcentagem de medicamentos prescritos pelo nome genérico em todas as 14 publicações avaliadas foi inconsistente, estando sempre abaixo do valor preconizado pela OMS, de 100% (31, 32). Este resultado não satisfaz a prática do uso racional de medicamentos, sugerindo a necessidade de novos estudos para melhor esclarecer a causa dessa falha. A prescrição de medicamentos pelo nome genérico é importante, uma vez que a prescrição médica pelo nome comercial ou fantasia pode resultar em erros de dispensação ou dificultar o acesso ao medicamento, caso a marca não esteja disponível nas farmácias da unidade (25, 33).

A porcentagem de consultas nas quais ocorre prescrição de antibióticos ficou dentro dos parâmetros estabelecidos pela OMS em sete publicações (13, 17, 18, 24, 25, 27, 28), ou seja, metade dos estudos que utilizaram indicadores de prescrição tiveram resultados que não atendem a prática do uso racional de medicamentos (31, 32). A preocupação da OMS com a prescrição de antibióticos vincula-se à necessidade de evitar o seu uso abusivo pelo custo elevado e pelo risco de disseminação e resistência bacteriana (10, 34). Porém, como as taxas de doenças infecciosas podem ser diferentes entre países, bem como sofrer influências sazonais, a necessidade de prescrever antibióticos pode variar e, portanto, as conclusões não podem ser generalizadas. Assim, a análise desse aspecto demanda amostragem sistemática e período de coleta anual para contornar este viés.

**TABELA 1.** Características das publicações incluídas na revisão sistemática sobre uso racional de medicamentos na Atenção Primária à Saúde, 2011 a 2016

Autor (ano/referência)	País do estudo	Nível de estudo	Local do estudo	Duração	Objetivo	Desenho	Amostragem	Nº Amostral	Indicadores utilizados	Conclusões sobre o uso racional de medicamentos
Dong et al. (2011/13)	China	Província	680 clínicas rurais de atenção primária de 40 municípios em 10 províncias do oeste da China ocidental.	3 meses	Avaliar os padrões de prescrição de medicamentos usando os indicadores da OMS.	Transversal retrospectivo	Conveniência	20 125 prescrições	- Prescrição	O estudo forneceu evidências de prescrição irracional de medicamentos. Todos os indicadores de prescrição apresentaram valores fora do recomendado pela OMS.
Vooss e Diefenthaeler (2011/14)	Brasil	Local	Farmácia de um centro de atenção primária no município de Getúlio Vargas, estado do Rio Grande do Sul.	4 meses	Avaliar as prescrições de medicamentos de acordo com os indicadores da OMS.	Transversal retrospectivo	Conveniência	1 030 prescrições	- Prescrição	O estudo forneceu evidências de prescrição irracional de medicamentos. A maioria dos indicadores de prescrição apresentou valores fora do recomendado pela OMS.
Oliveira et al. (2012/15)	Brasil	Local	Unidade de atenção primária do município de Salvador, estado da Bahia.	2 meses	Caracterizar o uso de medicamentos em uma unidade de saúde através dos indicadores da OMS.	Transversal, retrospectivo	Conveniência	1 230 prescrições	- Prescrição	O estudo forneceu evidências de prescrição irracional de medicamentos. A maioria dos indicadores de prescrição apresentou valores fora do recomendado pela OMS.
Souza et al. (2012/16)	Brasil	Local	Farmácia de atenção primária em um município do estado de Santa Catarina.	2 meses	Avaliar a demanda atendida a medicamentos e o perfil de prescrição através dos indicadores da OMS.	Transversal prospectivo	Conveniência	100 prescrições	- Prescrição	O estudo forneceu evidências de prescrição irracional de medicamentos. A maioria dos indicadores de prescrição apresentou valores fora do recomendado pela OMS.
Ahmed e Islam (2012/17)	Bangladesh	Província	30 complexos de saúde Upazila rurais e 20 clínicas urbanas na área da província de Dhaka.	2 meses	Avaliar a disponibilidade, o uso racional de medicamentos e a acessibilidade das unidades de atenção primária rurais e urbanas no país.	Transversal prospectivo	Conveniência e aleatória sistemática	1 496 encontros	- Prescrição - Assistência - Serviço	Os indicadores de prescrição, assistência e serviço revelaram práticas de uso irracionais. Todos os resultados ficaram fora dos valores recomendados pela OMS.
El Mahalli (2012/18)	Arábia Saudita	Província	10 centros de atenção primária na Província Oriental.	12 meses	Avaliar a racionalidade das prescrições de medicamentos através dos indicadores da OMS.	Transversal retrospectivo	Aleatória sistemática	1 000 prescrições	- Prescrição	O estudo forneceu evidências de prescrição irracional de medicamentos. Todos os indicadores de prescrição apresentaram valores fora do recomendado pela OMS, exceto a porcentagem de injetáveis.
El Mahalli et al. (2012/19)	Arábia Saudita	Província	10 centros de atenção primária na Província Oriental.	3 meses	Avaliar o desempenho dos centros de atenção primária utilizando os indicadores de assistência e serviço da OMS.	Transversal prospectivo	Aleatória sistemática	300 pacientes	- Assistência - Serviço	Os indicadores de assistência e serviço revelaram práticas irracionais. Todos os resultados apresentaram valores fora do recomendado pela OMS.
Rempel et al. (2013/20)	Brasil	Local	Unidade básica de saúde do município de Erechim, estado do Rio Grande do Sul.	2 meses	Avaliar a qualidade do atendimento em pacientes utilizando os indicadores desenvolvidos pela OMS.	Transversal prospectivo	Conveniência	200 prescrições	- Assistência	Os indicadores de assistência revelaram práticas irracionais. Todos os resultados foram abaixo do preconizado pela OMS.
Laste et al. (2013/21)	Brasil	Local	Centro de APS do município de Lajeado, estado do Rio Grande do Sul.	2 anos	Analisar os indicadores propostos pela OMS em prescrições médicas.	Transversal prospectivo	Conveniência	292 prescrições	- Prescrição	O estudo forneceu evidências de prescrição irracional de medicamentos. A maioria dos indicadores de prescrição apresentou valores fora do recomendado pela OMS.
Ferreira et al. (2013/22)	Brasil	Regional	Seis ambulatórios universitários de atenção primária em cidades das regiões Sul e Centro-Oeste.	3 anos	Investigar padrões de prescrição em diferentes níveis de atendimento de saúde.	Transversal prospectivo	Conveniência	1 956 Prescrições	- Prescrição - Serviço	Os indicadores de prescrição e de serviço forneceram evidências práticas de uso irracional de medicamentos. A maioria dos indicadores apresentou valores fora do recomendado pela OMS.
Díaz e Sánchez (2014/23)	Venezuela	Municipal	13 ambulatórios rurais de atenção primária no município de Atures, Estado do Amazonas.	3 meses	Descrever o uso de medicamentos em ambulatórios rurais, através dos indicadores de uso básico de medicamentos da OMS.	Transversal retrospectivo	Conveniência	1 238 prescrições	- Prescrição - Serviço	Os indicadores de prescrição e de serviço fornecem evidências práticas de uso irracional de medicamentos. A maioria dos indicadores apresentou valores fora do recomendado pela OMS.
Dourado e Rizzotto (2015/24)	Brasil	Local	Centro de atenção primária em um município no oeste do Estado do Paraná.	1 ano	Analisar a prática terapêutica de médicos (clínico geral e pediatra) e a qualidade da assistência farmacêutica.	Transversal prospectivo	Aleatória	200 encontros	- Prescrição - Assistência - Serviço	Os indicadores de prescrição, assistência e serviço revelaram práticas de uso irracionais. A maioria dos resultados obtidos ficou fora dos valores recomendados pela OMS.
Adisa et al. (2015/25)	Nigéria	Municipal	Oito centros de cuidados primários no município de Ibadan do Estado de Oyo, Sudoeste da Nigéria.	3 meses	Avaliar os padrões de prescrição e as opiniões dos pacientes sobre as práticas de cuidados de saúde.	Transversal prospectivo	Conveniência	400 prescrições	- Prescrição	O estudo forneceu evidências de prescrição irracional de medicamentos. Todos os indicadores de prescrição apresentaram valores fora do recomendado pela OMS.
Aravamuthan et al. (2016/26)	Índia	Distrito	Cinco farmácias comunitárias de diferentes cidades de um distrito do norte do estado de Tamil Nadu, sul da Índia.	1 ano	Avaliar os padrões de uso de medicamentos essenciais através de indicadores da OMS, acesso à saúde e indicadores complementares.	Transversal prospectivo	Aleatória sistemática	1 052 pacientes	- Prescrição - Assistência - Serviço	Os indicadores de prescrição, assistência e serviço revelaram práticas de uso irracionais de medicamentos. A maioria dos resultados obtidos ficou fora dos valores recomendados pela OMS.
Ahiabu et al. (2016/27)	Gana	Distrito	Duas clínicas (filantrópica e particular) e um centro de saúde (governamental) de atenção primária nos distritos de New- Juaben e Atiwa, na região oriental.	1 ano	Avaliar práticas de prescrição de antibióticos em ambientes de atenção primária usando os indicadores da OMS/INRUD e explorando fatores de influência.	Transversal retrospectivo	Aleatória	1 600 prescrições	- Prescrição	O estudo forneceu evidências de prescrição irracional de medicamentos. Todos os indicadores de prescrição apresentaram valores fora do recomendado pela OMS.
Yousif e Supakankunti (2016/28)	Sudão	Província	Centros de saúde primários (público, privado e outros) onde trabalham 197 clínicos gerais no estado de Gezira.	6 meses	Avaliar a qualidade da prescrição entre médicos de clínica geral em diferentes tipos de centros de saúde primários.	Transversal, retrospectivo	Aleatória sistemática	19 700 prescrições	- Prescrição	O estudo forneceu evidências de prescrição irracional de medicamentos. A maioria dos indicadores de prescrição apresentou valores fora do recomendado pela OMS.

**TABELA 2:** Síntese dos resultados da revisão sistemática sobre uso racional de medicamentos na Atenção Primária à Saúde, 2011 a 2016

Indicadores	Autor (ano/referência)^a^																Valores de referência
	Dong (2011/13)	Vooss (2011/14)	Oliveira (2012/15)	Souza (2012/16)	Ahmed (2012/17)	El Mahalli (2012/18)	El Mahalli (2012/19)	Rempel (2013/20)	Laste (2013/21)	Ferreira (2013/22)	Díaz (2014/23)	Dourado (2015/24)	Adisa (2015/25)	Aravamuthan (2016/26)	Ahiabu (2016/27)	Yousif (2016/28)	
***Prescrição***																	
No. médio de medicamentos prescritos por consulta	2,4	2,0	2,0	2,4	2,3	2,4	-	-	2,4	2,3	1,5	3,2	5,8	3,7	4,2	2,5	<2 ^b^
% genéricos	64,1	72,8	72,0	86,8	0,0	61,2	-	-	86,1	86,4	86,9	77,8	68,0	2,5	83,7	46,3	100 ^b^
% antibióticos	48,4	21,7	17,0	19,0	43,8	32,2	-	-	9,6	15,5	23,9	71,5	55,0	22,0	63,7	54,7	<30^b^
% injetáveis	22,9	2,4	21,5	3,0	Ausente	2,0	-	-	3,0	3,1	11,3	15,5	52,5	7,2	24,0	12,8	<20^b^
% medicamentos prescritos da lista local ou nacional	67,7	80,3	99	91,5	64,5	99,2	-	-	71,4	81,4	52,5	92,9	99,1	99,8	90,6	81,2	100^b^
***Assistência***	-	-	-	-	-	-	-	-	-	-	-	-	-	-	-	-	-
Tempo médio de consulta (min)	-	-	-	-	3,8	-	7,3	Ausente	-	-	-	6,13	-	< 11	-	-	≥15^c^
Tempo médio de dispensação (s)	-	-	-	-	90	-	100	81	-	-	-	99,6	-	≤ 600	-	-	≥180^c^
% realmente dispensados	-	-	-	-	60,1	-	99,6	85,8	-	-	-	90,2	-	99,8	-	-	100^b^
% medicamentos etiquetados	-	-	-	-	54,2	-	10,0	92,4	-	-	-	92,0	-	Ausente	-	-	100^b^
% de usuários que conhecem a dose correta	-	-	-	-	74,5	-	79,3	89,4	-	-	-	55,0	-	Ausente	-	-	100^b^
Serviço	-	-	-	-	-	-	-	-	-	-	-	-	-	-	-	-	-
% disponibilidade de cópias da LLME	-	-	-	-	51,0	-	90,0	-	-	Ausente	69,6	100	-	Ausente	-	-	100^b^
% disponibilidade de medicamentos chaves	-	-	-	-	10,5	-	59,2	-	-	68,1	48,7	90,2	-	99,8	-	-	100^b^
																	

a “-” significa que o autor não utilizou o grupo de indicadores. “Ausente” significa que o autor não apresentou resultado, embora tenha utilizado o grupo de indicadores de uma das três áreas.

b Dumoulin et al. (31); Harvard Medical School and Harvard Pilgrim Health, World Health Organization (WHO) (32).

c Bittner et al. (37).

**TABELA 3:** Propostas para a promoção do uso racional de medicamentos sugeridas por estudos incluídos na revisão sistemática sobre uso racional de medicamentos na Atenção Primária à Saúde, 2011 a 2016

Autor (ano/referência)	Propostas para melhorar o uso racional de medicamentos
Dong et al. (2011/13)	Sugerem que sejam realizados estudos de intervenção para avaliar modos de promover a prescrição racional de medicamentos. Por exemplo, um programa de treinamento educacional poderia ser conduzido e avaliado entre os médicos da aldeia para reduzir o uso irracional de drogas, em particular antibióticos e injetáveis.
Vooss e Diefenthaeler (2011/14)	Sugerem que, para ajustar o indicador percentual dos medicamentos prescritos com o nome genérico, os prescritores devam estar informados e principalmente que a LLME da cidade utilize o nome genérico.
Oliveira et al. (2012/15)	Sinalizam a necessidade de padronizar as condutas terapêuticas por meio de protocolos clínicos estabelecendo o uso racional da forma farmacêutica, sendo também necessário investir em campanhas informativas e na educação permanente direcionada aos profissionais prescritores da unidade de saúde. Além disso, propõem a inserção do profissional farmacêutico dentro da equipe multiprofissional, contribuindo com medidas para o consumo racional e seguro dos medicamentos.
Souza et al. (2012/16)	Sinalizam a necessidade de desenvolver estratégias que melhorem a gestão da Assistência Farmacêutica Municipal, uma vez que há problemas de demanda não atendida, mesmo daqueles medicamentos essenciais presentes na lista municipal, provavelmente devido a desabastecimento.
Ahmed e Islam (2012/17)	Sinalizam a necessidade de esforços combinados para motivar e treinar os profissionais de saúde e as profissões afins (por exemplo enfermeiros, paramédicos, profissionais alopáticos, distribuidores de medicamentos e fabricantes) sobre os benefícios da prescrição genérica e dos medicamentos essenciais da lista nacional, especialmente para os pobres. Sugere ainda que a polifarmácia e o uso excessivo ou abusivo de medicamentos, especialmente antibióticos, sejam desencorajados. Além disso, indicam a necessidade de fortalecer a capacidade reguladora de medicamentos da autoridade do país.
El Mahalli (2012/18)	Sugere que os médicos dos centros de APS tenham educação contínua sobre prescrição racional de antibióticos e motivação para prescrever medicamentos pelo nome genérico e da lista nacional. Enfatiza a necessidade de futuros estudos para investigar as razões por trás do uso irracional de drogas. Além disso, sugere o uso do centro de saúde mais bem classificado como referência para outros centros na região.
El Mahalli et al. (2012/19)	Recomendam que os tempos de consulta sejam mais longos e que as razões para os curtos tempos sejam investigadas. Os sistemas de rotulagem de fármacos precisam ser melhorados para incluir o regime de fármaco, o nome do doente e a dose de fármaco, bem como melhorar a disponibilidade de fármacos chave nos estoques dos centros de saúde. Além disso, sugerem o uso do centro de saúde mais bem classificado como referência para outros centros na região.
Rempel et al. (2013/20)	Sugerem que a LLME seja revisada para atender as necessidades prioritárias de atenção à saúde na maioria da população da região em estudo, além disso, que outros estudos sejam realizados nas demais unidades básicas de saúde para que seja possível avaliar a real situação do município.
Laste et al. (2013/21)	Sugerem que a prescrição seja vista como um documento terapêutico de alta relevância, pois apenas dessa forma será um instrumento efetivo para assegurar o uso racional de medicamentos, prevenindo erros de medicação e não adesão a tratamento. Os prescritores e dispensadores precisam estar cientes do seu papel e de sua responsabilidade, e as LLME precisam atender totalmente as necessidades da população. Além disso, apontam a necessidade de mais estudos com indicadores para o maior entendimento da realidade e para a elaboração de políticas e estratégias reorientadoras da Assistência Farmacêutica.
Ferreira et al. (2013/22)	Sinalizam que as práticas de prescrição de medicamentos devem ser melhoradas independentemente do nível de prestação de cuidados de saúde. Assim, faz-se necessário implementar diretrizes institucionais para a obtenção de padrões de prescrição mais adequados, promover a prescrição baseando-se na LLME e ressaltar a importância dessas práticas na escola de medicina e na educação médica continuada para o uso mais racional e seguro dos medicamentos.
Díaz e Sánchez (2014/23)	Sugerem que as autoridades de saúde conheçam e utilizem a LLME na APS, o que facilita a gestão relacionada ao medicamento. O conhecimento das características endêmicas e do perfil dos estabelecimentos de saúde pode contribuir para inclusão de medicamento na lista. Além disso, o acesso à informação atualizada e imparcial sobre as diretrizes terapêuticas pode promover a cada usuário o melhor tratamento, permitindo o uso racional dos recursos disponíveis, além da formação e supervisão dos profissionais de saúde, educação dos consumidores e o fornecimento de medicamentos apropriados em quantidades suficientes.
Dourado e Rizzotto (2015/24)	Sinalizam a necessidade de desenvolver ações direcionadas ao uso racional de medicamentos e redução do uso abusivo de antibióticos. Sugerem a criação de um programa de educação permanente em saúde aos prescritores e aos servidores envolvidos com a dispensação de medicamentos, visando à prescrição racional, bem como uma melhor qualidade na atenção farmacêutica prestada aos usuários. A adoção da listagem padronizada de medicamentos essenciais deve fazer parte da política de saúde do município, pois melhora a relação custo-benefício da prescrição. Sugerem ainda o desenvolvimento de pesquisas qualitativas junto aos profissionais do serviço de saúde, sobretudo aos prescritores para uma melhor avaliação dos fatores que possam estar influenciando o uso não racional de medicamentos.
Adisa et al. (2015/25)	Ressaltam a necessidade de treinamento regular e permanente sobre o uso racional de medicamentos aos trabalhadores da APS, especialmente de médicos e farmacêuticos, os quais precisam ser motivados e encorajados a praticar de forma a assegurar cuidados de saúde de qualidade para as pessoas.
Aravamuthan et al. (2016/26)	Ressaltam a necessidade de instituir uma diretriz, produzida pelos órgãos políticos regulatórios, para orientar os hábitos de prescrição de todos os clínicos do país. Sugerem ainda que a aplicação das diretrizes seja rigorosa e que haja monitoramento consistente para a efetiva adesão dos clínicos.
Ahiabu et al. (2016/27)	Ressaltam a necessidade de futuros inquéritos nacionais sobre o uso de antibióticos com estratégia de amostragem representativa que considera a diversidade nos tipos de instalações de saúde no país. De maior importância, sugerem a implementação de intervenções que visem ao diagnóstico e a gestão para lidar com o uso inadequado de antibióticos e promover o uso racional.
Yousif e Supakankunti (2016/28)	Sugerem que outros estudos sejam conduzidos para determinar os fatores que causam as discrepâncias consideráveis sobre a prática de prescrição irracional entre os médicos de clínica geral das unidades de saúde de propriedade do Fundo Nacional de Seguro de Saúde, Ministério da Saúde do Estado e outros (interesses privados, universidades e organizações não governamentais).

No indicador de porcentagem de consultas em que se prescrevem medicamentos injetáveis, quatro artigos (13, 15, 25, 27) descreveram cenários que não atenderam o uso racional de medicamentos por apresentarem valores > 20% (31, 32). Esse indicador também pode sofrer influências socioculturais, de sazonalidade e ainda de gestão farmacêutica, uma vez que a LLME pode conter formas farmacêuticas injetáveis, influenciando a prescrição. A preocupação da OMS com a prescrição de injetáveis também está vinculada ao uso abusivo e custo elevado, além do risco de reações difíceis de reverter ou potencialmente fatais.

Por fim, quanto à porcentagem de medicamentos prescritos presentes na LLME, todas as 14 publicações relataram valores abaixo do recomendado, que é de 100% (31, 32) das prescrições em harmonia com a política nacional de medicamentos. Dados semelhantes foram descritos anteriormente em países da Europa, (55,1%), Américas (71,4%) e Sudeste Asiático (81%) (35). O uso de medicamentos das LLME é importante pelos critérios que orientam a inclusão de medicamentos nessa lista, baseada na realização de testes, uso clínico estabelecido e custo mais baixo do que o de outros medicamentos (36).

### Indicadores da assistência ao paciente

Das quatro publicações (17, 19, 24, 26) que avaliaram o tempo médio dedicado pelo profissional médico aos pacientes no processo de consulta e prescrição, todas apresentaram valores abaixo dos 15 minutos mínimos preconizados pela OMS (10, 37). Este resultado não satisfaz a prática do uso racional de medicamentos, uma vez que o tempo insuficiente de consulta pode levar a diagnóstico inadequado e interação médico-paciente insuficiente (30). Em relação ao tempo médio de dispensação entrega do medicamento ou produto para saúde, realizada pelo profissional farmacêutico, promovendo condições para que o paciente utilize o medicamento ou produto da melhor maneira possível (38) das cinco publicações que utilizaram este indicador, quatro (17, 19, 20, 24) ficaram abaixo do tempo mínimo de 180 segundos (10, 37).

A porcentagem de medicamentos prescritos que são efetivamente dispensados determina a capacidade dos serviços de saúde de proporcionar os medicamentos (10). Na presente revisão, nenhuma das cinco publicações avaliadas neste quesito atendeu a prática do uso racional de medicamentos, que recomenda a dispensação de 100% dos medicamentos prescritos.

Esse fato é inquietante, pois a prescrição de medicamentos fora da LLME e a falta de medicamentos essenciais nos serviços de saúde comprometem não só o acesso regular a medicamentos como também a segurança dos pacientes que dependem de seu uso continuado, especialmente aqueles de menor poder aquisitivo (39). Dos medicamentos em estoque nas unidades de saúde, a porcentagem de medicamentos corretamente etiquetados também não satisfez o uso racional de medicamentos. De acordo com a OMS, cada rótulo deve conter o regime de administração, o nome do paciente e a dose utilizada (10). Nesse caso, a prática de rotulagem foi muito carente, uma vez que todas as quatro publicações que utilizaram este indicador (17, 19, 20, 24) apresentaram resultados abaixo dos 100% recomendados (31, 32). Isso pode ser atribuído à falta de um sistema de rotulagem, à carência de recursos financeiros, à ausência de farmacêutico na equipe de saúde e à deficiência de gestão e política de assistência farmacêutica. Por outro lado, pode-se questionar se de fato a ausência de um sistema de rotulagem nas unidades de APS afastaria a prática do uso racional dos medicamentos. Vale ressaltar que o fracionamento de medicamentos demanda um sistema de rotulagem, porém essa prática encontra-se mais presente na atenção terciária de saúde. Assim, poderíamos sugerir que a aplicação desse indicador na APS deveria ser condicionada à presença do serviço de fracionamento.

Por fim, quanto à porcentagem de usuários que conhecem a dose correta de seus medicamentos, observou-se que nenhuma das publicações atendia a prática do uso racional de medicamentos, com todos os quatro estudos apresentando resultados abaixo dos 100% recomendados (31, 32). Este fato reflete a ineficácia das informações fornecidas aos usuários sobre a posologia dos seus medicamentos nas unidades de APS. Dessa forma, o pouco conhecimento do usuário sobre como utilizar seus medicamentos pode resultar da baixa qualidade das consulta e da dispensação; essas limitações podem aliar-se ao baixo grau de escolaridade, idade avançada dos pacientes e ausência de profissional farmacêutico.

### Indicadores de serviço

Uma cópia da LLME na APS não estava disponível em três dos quatro estudos que utilizaram indicadores de serviço nesta revisão (17, 19, 23). O acesso à LLME contribui para nortear os prescritores a respeito dos medicamentos padronizados e melhorar a relação custo-benefício da prescrição. Além disso, a LLME auxilia na gestão da assistência farmacêutica para fins de abastecimento e acessibilidade aos medicamentos. Portanto, qualquer valor diferente de 100% pode ser considerado insatisfatório para o uso racional de medicamentos. Em relação à disponibilidade dos medicamentos essenciais categorizados como de referência ou chave nos serviços de saúde, todos os seis estudos que avaliaram esse quesito (17, 19, 22-24, 26) apresentaram valores abaixo do recomendado (31, 32).

A análise dos resultados desses indicadores possibilita reflexões sobre as possíveis causas dos problemas encontrados na assistência farmacêutica da APS. Oliveira et al. (40) sugerem, dentre possíveis causas, falta de comprometimento ou de ingerência do gestor de saúde em relação à assistência farmacêutica, escassez de recursos financeiros, ausência de planejamento e programação para a aquisição de medicamentos, aquisições equivocadas e armazenamento em condições inapropriadas, o que contribui para a deterioração dos medicamentos, ocasionando perdas.

### Pontos fortes e limitações

Um ponto forte deste estudo foi o uso de uma metodologia de revisão sistemática de qualidade e a utilização de cinco bases de dados diferentes. Além disso, estudos com os indicadores de uso de medicamentos da OMS/INRUD são atualmente escassos, e esta revisão, além de estimular novas publicações, torna públicas propostas para melhorar o uso racional de medicamentos que podem servir como base para possíveis estudos interventivos. A inclusão de publicações com foco apenas na APS confere outro ponto forte, uma vez que os indicadores foram idealizados para serem aplicados nesse nível de atenção.

As publicações foram selecionadas para o estudo a partir apenas dos achados nas bases de dados eletrônicas citadas, sem busca manual em outras fontes, como listas de referências dos artigos selecionados e contato com pesquisadores para identificar estudos não publicados. Além disso, o estudo ficou limitado a artigos publicados em inglês, espanhol ou português. Outra limitação foi a falta de estudos com esses indicadores em países desenvolvidos, o que restringiu a generalização dos dados. Para esse fato pode-se pressupor a não utilização de uma base de dados principalmente de origem europeia.

É importante ressaltar que a maioria das publicações (56,3%) teve amostragem não probabilística por conveniência; portanto, os resultados não puderam ser generalizados estatisticamente para a população da pesquisa, e as técnicas de inferência estatística utilizadas possuem validade qualitativa.

A presente revisão sistemática não incluiu uma metanálise. Embora isso não seja necessariamente uma condição limitante, idealmente deveria haver uma certa uniformidade entre os estudos incluídos em relação ao método de coletas de dados. Entretanto, esse aspecto não foi considerado para a análise. Além disso, as publicações relataram cenários de sistemas de saúde distintos, inseridos em contextos com diferentes valores culturais. Por fim, esses indicadores básicos não mostram as causas dos problemas, apenas os identificam, bem como não conseguem determinar se os medicamentos prescritos foram efetivamente utilizados pelos usuários.

## CONCLUSÃO

A capacidade dos serviços de APS, nos diferentes países, foi insuficiente para apoiar o uso racional de medicamentos. Em geral, os estudos, através dos indicadores de prescrição, assistência e serviço, mostraram valores não satisfatórios em relação aos preconizados como ideais pela OMS. Assim, revelaram práticas de uso irracional de medicamentos. Por essa razão, são necessários novos estudos. Ao mesmo tempo, há espaço para propor atualizações nos valores de referência dos parâmetros de uso racional de medicamentos, assim como inclusão ou exclusão de indicadores com novas validações. Principalmente, é importante considerar a realização de estudos intervencionistas que apresentem melhorias para o uso racional de medicamentos.

Em termos básicos, para que ocorra o uso racional de medicamentos, é necessário que os países possuam uma política nacional de medicamentos implantada, que fortaleça as autoridades reguladoras de medicamentos e a gestão da assistência farmacêutica. Dessa implantação partem três bases importantes que precisam ser aperfeiçoadas: a lista de medicamentos essenciais do país; a gestão e a operacionalização dos medicamentos; e os protocolos clínicos e diretrizes terapêuticas. A partir dessas ferramentas partem as estratégias interventivas para melhorias de produto, serviço e processo.

## Agradecimentos.

À coordenação e ao Programa de Pós-Graduação em Ciências Farmacêuticas (PPGCF) da Universidade Federal de Pernambuco (UFPE) pelo incentivo a estudos relacionados à utilização de medicamentos e saúde pública.

## Declaração

As opiniões expressas no manuscrito são de responsabilidade exclusiva dos autores e não refletem necessariamente a opinião ou política da RPSP/PAJPH ou da Organização Pan-americana de Saúde (OPAS).
